# Novel Antibody against a Glutamic Acid-Rich Human Fibrinogen-Like Protein 2-Derived Peptide near Ser91 Inhibits hfgl2 Prothrombinase Activity

**DOI:** 10.1371/journal.pone.0094551

**Published:** 2014-04-11

**Authors:** Wen-Zhu Li, Jue Wang, Rui Long, Guan-Hua Su, Dinesh-Kumar Bukhory, Jing Dai, Nan Jin, Shi-Yuan Huang, Peng Jia, Ting Li, Chen Fan, Kun Liu, Zhaohui Wang

**Affiliations:** 1 Department of Cardiology, Institute of Cardiovascular Disease, Union Hospital, Tongji Medical College, Huazhong University of Science and Technology, Wuhan, China; 2 Department of Hematology, Tongji Hospital, Tongji Medical College, Huazhong University of Science and Technology, Wuhan, China; 3 Department of Geriatrics, Institute of Geriatrics, Union Hospital, Tongji Medical College, Huazhong University of Science and Technology, Wuhan, China; Thomas Jefferson University, United States of America

## Abstract

Fibrinogen-like protein 2 (fgl2) is highly expressed in microvascular endothelial cells in diseases associated with microcirculatory disturbances and plays a crucial role in microthrombosis. Previous studies have demonstrated that the Ser89 residue is a critical site for mouse fgl2 prothrombinase activity. The aim of this study was to investigate the prothrombinase inhibitory ability of antibodies against an hfgl2-derived peptide. The peptide was termed NPG-12 because it is located at the N-terminus of membrane-bound hfgl2, contains 12 amino acid residues (corresponding to residues 76 to 87), and is rich in Glu. This peptide was selected as an antigenic determinant to produce antibodies in immunized rabbits using the DNAStar and HomoloGene software program. Abundant hfgl2 expression was induced in human umbilical vein endothelial cells through treatment with TNF-α. The generated anti-NPG-12 antibodies specifically recognize fgl2, as determined by ELISA, Western Blot and immunostaining. Moreover, one-stage clotting and thrombin generation tests provide evidence that the antibodies can reduce the hfgl2 prothrombinase activity without affecting the platelet-poor plasma prothrombin time (PT) or the activated partial thromboplastin time (APTT). In addition, the antibodies exerted undetectable influence on the proliferation or activation of bulk T cell populations. In conclusion, the selected peptide sequence NPG-12 may be a critical domain for hfgl2 prothrombinase activity, and the development of inhibitors against this sequence may be promising for research or management of hfgl2-associated microcirculatory disturbances.

## Introduction

Increasing evidence based on immunohistochemistry demonstrates that fibrinogen-like protein 2 (fgl2) is abundantly expressed in microcirculatory disturbances, such as in hepatic sinusoidal endothelial cells associated with human viral hepatitis [1]–[3], graft microvascular endothelial cells in allograft and xenograft rejection [4], uterus trophoblast and decidua in cytokine-induced fetal loss syndrome [5], tumor microvessel endothelium [6], and cardiac microvascular endothelial cells in type 2 diabetes or cardiac ischemia/reperfusion injury [7]–[9]. These conditions are characterized by fibrin deposition or microthrombus formation in microvascular endothelial cells. Particularly, membrane-bound fgl2 expressed on microvascular endothelial cells can directly activate prothrombin to form fibrin deposits *in situ* and mediate microthrombosis independent of the classic intrinsic and extrinsic coagulation pathways [10], [11].

**Figure 1:**
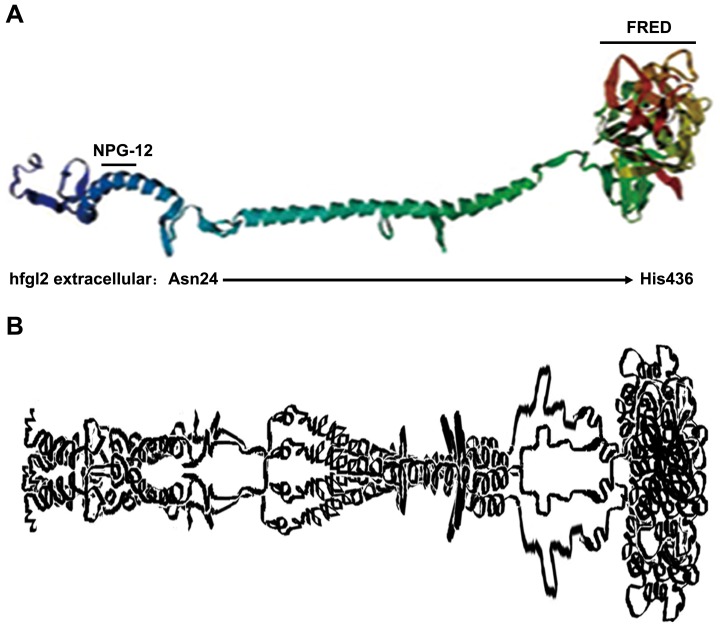
ModBase-MODEL prediction of the hfgl2 structure. (A) Prediction of hfgl2 protein structure was obtained from ModBase-MODEL website, and A4D1B8 was selected as the primary database link (http://modbase.compbio.ucsf.edu/modbase-cgi/search_form.cgi). NPG-12 and FRED domain are indicated by black bars. (B) Schematic illustration of tetrameric hfgl2 protein adapted from ModBase-MODEL analysis.

Fgl2, a 65-kD protein belonging to the fibrinogen superfamily, has been proven to be a multifunctional protein (Fig. 1) [2], [10], [12], [13]. The fgl2 gene was cloned from human peripheral blood T lymphocytes, and data had suggested that the secreted fgl2 protein presented as tetramer in the culture supernatant lacked coagulation activity (Fig. 1B) [14], [15]. The human and murine fgl2 proteins share 78% overall identity with a greater conservation at the C-terminus, which is a region referred to as the fibrinogen-related domain (FRED) (Fig. 1A). This domain exhibits immunomodulatory activity and lacks prothrombinase activity, as indicated by several lines of evidence [12], [16]. Fgl2 is also defined as a serine protease based on observation that its prothrombinase activity can be inhibited by diisopropylfluorophosphate (DFP), a specific serine protease inhibitor [10], [17]. There are three Ser-Xaa-Xaa-Lys (SXXK) motifs in mouse fgl2 (mfgl2) that can be catalyzed by serine peptidase clan E. Interestingly, the mutation of Ser135 or Ser425 to alanine does not alter mfgl2 prothrombinase activity [10], [13], whereas the Ser89 residue is demonstrated to be critical for the prothrombinase activity of mfgl2 by the results of site-directed mutagenesis of the Ser89-Xaa-Xaa-Lys motif [10]. However, the human fgl2 (hfgl2) domain that acts as a serine prothrombinase is not well studied.

Our knowledge of hfgl2 as a serine prothrombinase makes it possible to create inhibitors against hfgl2 prothrombinase activity. In the fgl2 coagulation cascade, prothrombin can be activated by membrane-bound fgl2, which requires phospholipids/cell membranes, calcium, and factor Va for its full activity [10]. Sequence analyses indicate the existence of an hfgl2 SXXK motif near Ser91, which is similar to mfgl2 Ser89 [10], [12]. Thus, we hypothesized that residues near hfgl2 Ser91 resembling FXa may also contain a domain rich in glutamic acids (Glu) that facilitates Ca^2+^ binding. In this study, we generated novel polyclonal antibodies against an hfgl2 peptide termed NPG-12. This name was chosen due to the peptide's location at the N-terminus of membrane-bound hfgl2, its length of 12 amino-acid residues (corresponding to residues 76^th^-87^th^, near Ser91), and its abundance of Glu residues. Further experiments investigated the effects of antibodies targeting this peptide on the hfgl2 protein function, focusing mainly on the inhibition of prothrombinase activity in human umbilical vein endothelial cells (HUVECs) exposed to TNF-α and the potential off-target immunomodulatory effects on T lymphocytes.

## Materials and Methods

### Ethics statement

In this study, human umbilical vein endothelial cells (HUVECs) obtained from Promocell (Heidelberg, Germany) conform to the principles expressed in the Declaration of Helsinki. After obtaining written informed consent from individual donors, human purified peripheral blood T cells were obtained from healthy volunteers following protocols approved by Huazhong University of Science and Technology, Tongji Medical College, Wuhan Union Hospital Clinical Research Ethics Board (Approval ID:S146). Animal experiments were carried out in strict accordance with the recommendations in the Guide for Care and Use of Laboratory Animals of the National Institutes of Health. And the protocol was approved by the Ethical Committee on Animal Experiments of Tongji Medical College, Huazhong University of Science and Technology (Approval ID: 00011646). All surgery was performed under sodium pentobarbital anesthesia, and efforts were made to minimize suffering [18].

### Antigen preparation

Based on the locations of conserved Glu residues in hfgl2 sequence shown by HomoloGene and DNAStar and its nature as a serine protease, we identified a peptide NPG-12 that was named for its location at the N-terminus of membrane-bound hfgl2, its length of 12 amino acid residues (corresponding to residues 76–87), and its abundance of Glu residues [19], [20]. NPG-12 scrambled peptide (NSP) comprised of the same amino acids but in different order as NPG-12 was synthesized as a control. The peptides coupling with cysteine (Cys) was synthesized by GL Biochem Ltd (China) through solid-phase chemistry (PSSM-8, Shimadzu, Japan), and high-pressure liquid chromatography (LC-20A, Shimadzu, Japan) was utilized to examine its purity. KLH (Sigma, USA) was selected as a conjugate, and Cys cross-linked with sulfo-SMCC-KLH (Pierce Chemical, USA) through a mercapto group [21]. Then, the peptide-KLH was used as an antigen [18], [20]–[23].

### Antibody generation

Twenty four male New Zealand white rabbits (Laboratory Animal Center of Tongji Medical College, Wuhan, China) were divided into four groups (six rabbits per group). One group was immunized with 500 µg of NPG-12 linked to KLH (NPG-12-KLH), one group was immunized with 500 µg of NSP linked to KLH (NSP-KLH), one group was injected with KLH in PBS (PBS-KLH) as a control, and the other group was used as the normal control without injection. The NPG-12-KLH, NSP-KLH and PBS-KLH groups were emulsified with Complete Freund's adjuvant (Sigma, USA) through subcutaneous injection at multiple sites on the 1^st^ day. The antibodies were boosted with 250 µg of NPG-12-KLH, NSP-KLH or PBS-KLH emulsified with incomplete Freund's adjuvant (Sigma, USA) on the 14^th^, 28^th^, 42^nd^, and 56^th^ days. Blood samples were collected from the marginal ear vein before injection. On the 63^rd^ day, we collected blood samples from the carotid artery. The titers of the antisera were screened using ELISA. And the antisera were preliminary purified through 50%, 40% to 33% ammonium sulfate saturation [24], [25]. Antibodies obtained from ammonium sulfate precipitation were purified on a protein A column (Bio-Rad, US). After additional purification by a NPG-12 peptide-affinity column (GL Biochem Ltd, China), the antibodies were adjusted to the concentration of 2 mg/ml by Millipore Centriplus [18], [25], [26].

### Cell culture and siRNA transfection

HUVECs obtained from Promocell (Heidelberg, Germany) were cultured in M199 Medium (Gibco, USA) at 37°C in 5% CO_2_. The medium was supplemented with 5% fetal bovine serum (Hyclone, USA), 2 mmol/L glutamine, and 1% penicillin/streptomycin (Hyclone, USA). Subculture cells were obtained by treating the cells with 0.25% trypsin-0.01% EDTA solution (Hyclone, USA). The cells used were between passages 2 and 6 [27], [28]. After 12 h of starvation in serum-free M199 medium, the cells were treated with 50 ng/ml TNF-α (PeproTech, USA) for 24 h to induce high hfgl2 expression [29]. The fgl2 siRNA and scrambled siRNA (Ribobio, China) were transfected with Lipo2000 (Invitrogen, USA) according to the manual of the manufacturer [30]. For siRNA knock down assay, the HUVECs were incubated with transfection mixture in serum-free media for 8 h before changing fresh media and adding 50 ng/ml TNF-α to induce high hfgl2 expression. Then, the cells were obtained for another 72 h before harvest. Targeted mRNA sequence is 5′-CTAACGAGTTTCTCAAATA-3′. The fgl2 siRNA sense sequence is 5′-CUAACGAGUUUCUCAAAUAdTdT-3′, and the anti-sense sequence is 5′-UAUUUGAGAAACUCGUUAGdTdT-3′ [31], [32].

### ELISA

Microtiter plates were coated with NPG-12, NSP or KLH at a final concentration of 10 µg/ml and blocked with BSA. Serially diluted 100 µl antiserum was added into wells, and HRP-conjugated goat anti-rabbit IgG (1∶12000) (Sigma, USA) was used as a secondary antibody. Each well was added with 100 µl TMB/H_2_O_2_ (Boster, China) and incubated at 37°C in dark for 30 minutes, and then 100 µl 2 M H_2_SO_4_ was added into wells as a stop solution. The optical density (OD) was read at 450 nm, and all of the tests were conducted in triplicate [25], [33].

### Western Blotting

M-PER (Mammalian Protein Extraction Reagent; Thermo, USA) and a complete protease and phosphatase inhibitor cocktail (Roche, Switzerland) were added to culture flasks. The cells were then scraped off and shifted to centrifuge tubes. The supernatant whole-cell extracts were obtained, and the BCA™ Protein Assay Kit (Pierce, USA) was used to determine the protein concentration. The weights of the protein samples were adjusted to 35 µg, and the proteins were then denatured at 100°C and subjected to electrophoresis on 8% SDS-PAGE. The proteins were transferred from gels to nitrocellulose membranes (Invitrogen, USA), and the membranes were blocked with 5% nonfat dry milk. The proteins were incubated with anti-NPG-12 antibodies (1 µg/ml) and anti-NSP antibodies (1 µg/ml) overnight at 4°C, and a commercially available anti-fgl2 antibody (2 µg/ml, Abnova, Taiwan) was used as a positive control. HRP-conjugated (Sigma, USA) antibodies were used as secondary antibodies. The specific bands were visualized using the ECL reagent (Pierce, USA) and recorded by the Molecular Imager ChemiDoc XRS System (Bio-Rad, USA) [34].

### Immunostaining

HUVECs resuspended at a density of 5*10^5^ cells/ml were cultured in a confocal dish (Theromo, USA) for 48 h, synchronized for 12 h with serum-free M199 media, and stimulated with 50 ng/ml TNF-α for 24 h to induce the high expression of hfgl2. The cells were fixed with 4% paraformaldehyde (Sigma, USA), permeabilized with 0.1% triton X-100 (Sigma, USA), and then incubated with anti-NPG-12 antibodies diluted 1∶400 (5 µg/ml) or anti-fgl2 antibody diluted 1∶200 (5 µg/ml) overnight at 4°C before incubation with Cy5-labeled secondary antibody (Sigma, USA) at room temperature for 1 h in the dark [35]. The nucleus was stained with DAPI (Boster, China). The cells were observed using an Olympus FluoView™ FV1000 (Olympus, Japan) laser scanning confocal microscope [18].

### One-stage clotting assay

The cells were washed three times with RPMI1640 and treated with 0.25% trypsin-0.01% EDTA solution. The cells were then resuspended at a density of 6×10^6^ cells/ml and freeze-thawed three times to obtain the maximal total cellular procoagulant activity. Briefly, 100 µl of the HUVECs samples were mixed with 100 µl of FX, FII or FVII-deficient and normal plasma respectively (STAGO, France) and 100 µl of 25 mM CaCl_2_ (all of the reagents were prewarmed to 37°C). The clotting time was determined by the appearance of a fibrin clot [10]. The results were compared with the standard curve generated by serially diluted standard rabbit brain thromboplastin (Sigma, USA): a concentration of 36 µg/ml equals 100,000 mU [2], [10], [36].

### Thrombin generation test

HUVECs were washed three times with PBS and treated with 0.25% trypsin-0.01% EDTA solution. The cells were then resuspended at a density of 2×10^7^ cells/ml. A total of 2×10^5^ cells (10 µl) with different concentrations of anti-NPG-12 antibodies were mixed with 70 µl of human Xa-deficient plasma or mixed plasma, and 20 µl of platelet-poor plasma or 20 µl of a thrombin calibrator was then added to the mixture. After the mixture was incubated for 10 min at 37°C, CaCl_2_ and fluorogenic substrate (FluCa kit) were added to the mixture according to the procedure detailed by the manufacturer's instructions for the generation of calibrated automated thrombograms. The thrombin generation was evaluated based on its fluorescence, which was detected using a Fluoroskan Ascent microplate reader (Thermo, USA) [29], [37], [38]. The reagents were all obtained from STAGO (France).

### Prothrombin time (PT) and activated partial thromboplastin time (APTT)

Different concentrations of anti-NPG-12 antibodies were added to human platelet-poor plasma, and the mixture was incubated for 2 h at room temperature. The PT assay was initiated by rabbit brain powder, and APTT assay was initiated by the addition of cephalin. Normal platelet-poor plasma was used as a negative control, and heparin was added as a positive control. All samples were tested by the Department of Clinical Laboratory of Union Hospital using a STAR evolution coagulation analyzer (STAGO, France), and all of the reagents were obtained from STAGO. The assays were conducted in triplicate [39].

### Combination between anti-NPG-12 antibodies with Vitamin K-dependent coagulation factors

Combination between anti-NPG-12 antibodies with Vitamin K-dependent (VKD) coagulation factors (1 µg/lane) were evaluated by Western Blotting as detailed above [34]. Anti-NPG-12 antibodies combination with hfgl2 expressed by HUVECs was used as a positive control. Purified human prothrombin (Factor II), Factor X, Protein C and Protein S were obtained from Stago Inc. (France). Purified human Factor VII and Factor IX were obtained from Sino Biological Inc. (China), and human protein Z was obtained from Abcam Inc. (USA).

### T cell proliferation and activation

Purified human peripheral blood T cells were labeled with 1 µM CFSE (Invitrogen, USA), and 2×10^5^ cells per well were seeded in a 96-well round-bottom plate. The antibodies produced were diluted and added to the cultured medium to obtain final concentrations of 1∶20, 1∶100, and 1∶500 to observe their effect on human lymphocyte proliferation. In the T cell inhibition test, the 96-well plate was precoated with anti-CD3/CD28 monoclonal antibodies (2 µg/ml) and then incubated with the diluted anti-NPG-12 antibodies at final concentrations of 1∶20, 1∶100, and 1∶500. After 72 h of stimulation, the proliferated CD4^+^ and CD8^+^ T cells in each group were measured using flow cytometry (FCM) [40]–[42]. The FCM reagents were obtained from eBioscience. The levels of IL-2, IFN-γ, IL-4, IL-10 and secreted fgl2 in the culture supernatants were quantified by ELISA [40]–[43] using ELISA kits that were obtained from R&D Company, and all of the assays were conducted in triplicate.

### Statistical Analyses

The data are indicated as the means ± S.E.M. All of the statistical analyses were performed using GraphPad Prism V5.0 (GraphPad Software, San Diego, CA, USA). The significance of differences between selected groups was evaluated using one-way analysis of variance followed by a Bonferroni's post hoc analysis [44], [45].

## Results

### Generation of anti-NPG-12 antibodies

**Figure 2:**
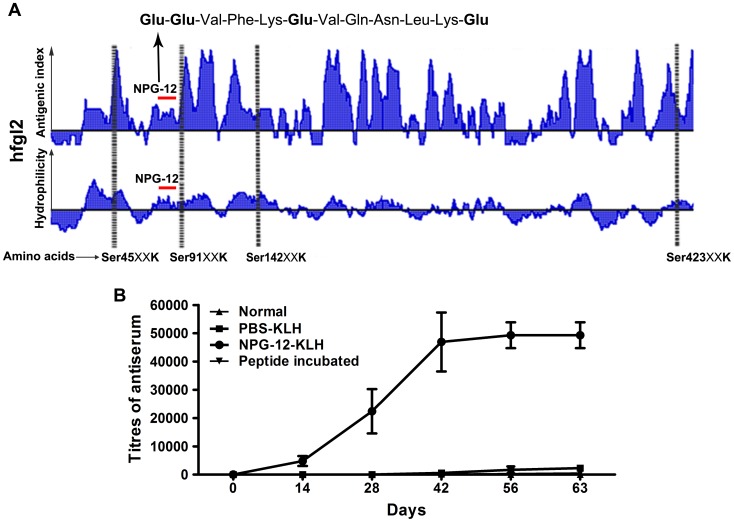
NPG-12 peptide selection and generation of anti-NPG-12 antibodies. (A) hfgl2 antigenic index (Jameson-Wolf) and hydrophilicity plot (Hopp-Woods) analysis. Four serine peptidase Clan E catalyzing sites (SXXK motifs) were identified. The target peptide NPG-12 mapped to positions 76 to 87 near Ser91XXK is indicated by a red bar. (B) The titer of the anti-NPG-12 antibodies were increased to its peak in the NPG-12-KLH group after the third immunization, whereas no detectable titer was found in the normal and the PBS-KLH and peptide-pre-incubated groups, as determined by ELISA. Peptide incubated: peptide NPG-12 pre-incubated with anti-NPG-12 antisera group.

The hfgl2 NPG-12 peptide containing 12 amino acids was chosen due to its location proximity to Ser91, its abundance of Glu residues, and its acceptable hydrophilicity and antigenicity using the DNAStar and HomoloGene software (Fig. 2A). The NPG-12 and NSP sequences are as following: H-Cys-**Glu**-**Glu**-Val-Phe-Lys-**Glu**-Val-Gln-Asn-Leu-Lys-**Glu**-OH and H-Cys-Phe-Glu-Val-Glu-Gln-Leu-Lys-Glu-Val-Asn-Lys-Glu-OH. The purity of NPG-12 and NSP were found to be ≥95% by high-pressure liquid chromatography. By immunizing rabbits with NPG-12-KLH, we generated polyclonal antibodies against hfgl2 NPG-12 with a high titer. Peptide was precoated as an antigen, and titers of the antisera were detected by indirect ELISA. Anti-NPG-12 antibody titers were markedly boosted and reached their peak (1∶51,200) after three immunizations, and these titers were maintained until terminal immunization in the NPG-12-KLH group (Fig. 2B). The titers in NSP group reached to 1∶25,600.

### Specificity of anti-NPG-12 antibodies

The polyclonal antibodies from the immunized rabbits were separately prepared from antiserum through ammonium sulfate precipitation and immunoaffinity chromatography. The purified anti-NPG-12 antibodies were proposed to specifically recognize NPG-12, while the antibodies recognizing linkers NSP or carrier protein KLH would not bind to the NPG-12 peptide-affinity column. The concentrations of the antibodies were adjusted to 2 mg/ml in PBS.

It has been reported that human TNF-α induced pig fgl2 expression and enhanced activation of human prothrombin in cultured porcine aortic endothelial cells (PAECs) [29]. Our group also demonstrated that TNF-α induced high rat fgl2 expression in cultured rat cardiac microvascular endothelial cells (RCMVEs) [7]. Thus, 50 ng/ml TNF-α was used as an inductive reagent to induce high hfgl2 expression in HUVECs. To confirm whether the anti-NPG-12 antibodies prepared were antigen-specific, we observed the specific recognition of peptide NPG-12 by ELISA, hfgl2 protein by western blotting and their binding to hfgl2 expressed in HUVECs by immunostaining.

**Figure 3:**
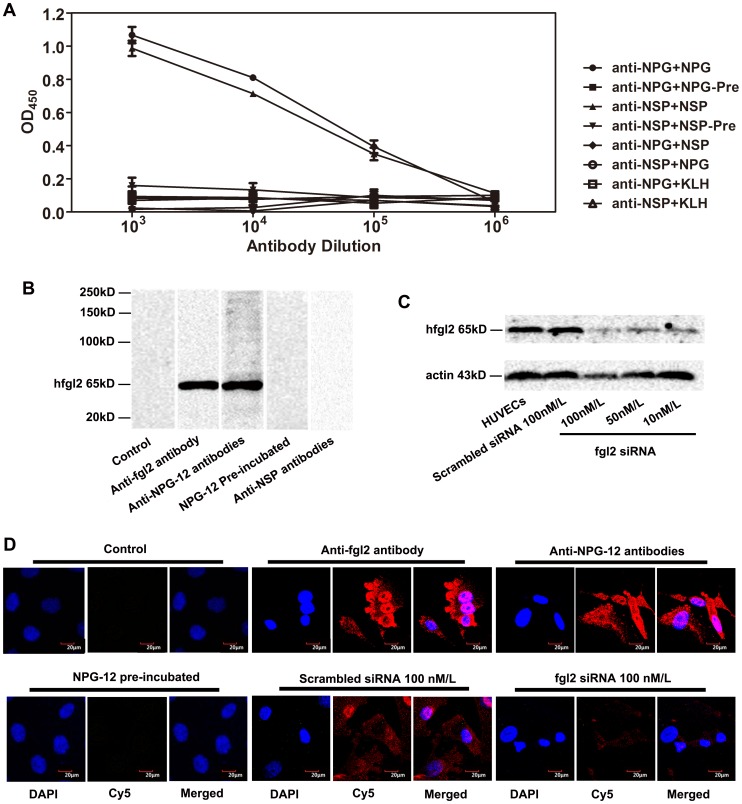
Specificity of anti-NPG-12 antibodies. (A) Peptides were precoated on ELISA plates, and probed with purified antibodies. Anti-NPG-12 antibodies only recognized NPG-12 and anti-NSP antibodies only recognized NSP. The values in the graph are the means ± S.E.M (n = 3). (B) Anti-NPG-12 antibodies specifically recognized hfgl2 expressed in HUVECs exposed to TNF-α for 24 h, as evidenced by western blotting. Anti-fgl2 antibody (2 µg/ml) and anti-NPG-12 antibodies (1 µg/ml) specifically recognized a 65-kD protein, and there was no detection in the control (no primary antibody), NPG-12-pre-incubated, or anti-NSP antibody groups. (C) WB of HUVECs lysates 72 h after siRNA transfection, gray-scale value or fgl2 expression was reduced by siRNA transfection dependent on fgl2-siRNA concentrations. Actin served as a loading control. (D) Representative HUVEC sections illustrate that the anti-NPG-12 antibodies specifically recognized hfgl2 on HUVECs exposed to TNF-α for 24 h or siRNA transfected HUVECs, as evidenced by immunostaining. The nuclei were stained with DAPI (blue), and the plasma membranes were stained with Cy5-labelled (red) secondary antibody in the anti-fgl2-antibody-treated (5 µg/ml) and anti-NPG-12-antibodies-treated (5 µg/ml) groups. The fluorescence intensity obviously decreased in fgl2-siRNA transfection group incubation with anti-NPG-12 and Cy5-labelled antibodies. No fluorescence was detected in the control group (no primary antibody) or the NPG-12-pre-incubated group treated with anti-NPG-12 antibodies. Scale bar  = 20 µm.

The results of indirect ELISAs were shown in Fig. 3A. The purified antibodies had high binding affinity to the precoated antigen peptide, while little or no binding affinity was observed when tested with other peptide or protein, indicating that these antibodies specifically recognized the NPG-12 or NSP. Whereas the antibodies neither binding with KLH-Cys-Peptide linkage nor contaminants from two-step antibody purification procedure was above background level.

The exposure to 50 ng/ml TNF-α led to marked expression of the hfgl2 protein in HUVECs, as shownin PAECs and RCMECs previously [7], [29]. As a positive control, anti-fgl2 antibody recognized a 65-kD band. The anti-NPG-12 antibodies generated in this study were also able to recognize the fgl2 protein. In contrast, there was no detection of IgGs in the NPG-12-pre-incubated or anti-NSP antibodies gruop (Fig. 3B). The gray-scale value of the 65 kD-band decreased when different concentrations of siRNA were used to knock down the hfgl2 expression (Fig. 3C). The binding of secondary structure of hfgl2 was detected by HUVECs exposed to 50 ng/ml TNF-α. The binding of anti-NPG-12 antibodies or anti-fgl2 antibody could be observed in HUVECs, and the fluorescence intensity decreased in fgl2-siRNA transfected HUVECs. In contrast, no HUVECs in the NPG-12-pre-incubated group were stained by IgGs (Fig. 3D).

### Anti-NPG-12 antibodies inhibit hfgl2 prothrombinase activity without affecting PT or APTT

Because fgl2 plays a crucial role in the coagulation pathway of fibrin deposition, we evaluated the prothrombinase activity of hfgl2 and the effects of the anti-NPG-12 antibodies as specific inhibitors on coagulation.

**Figure 4:**
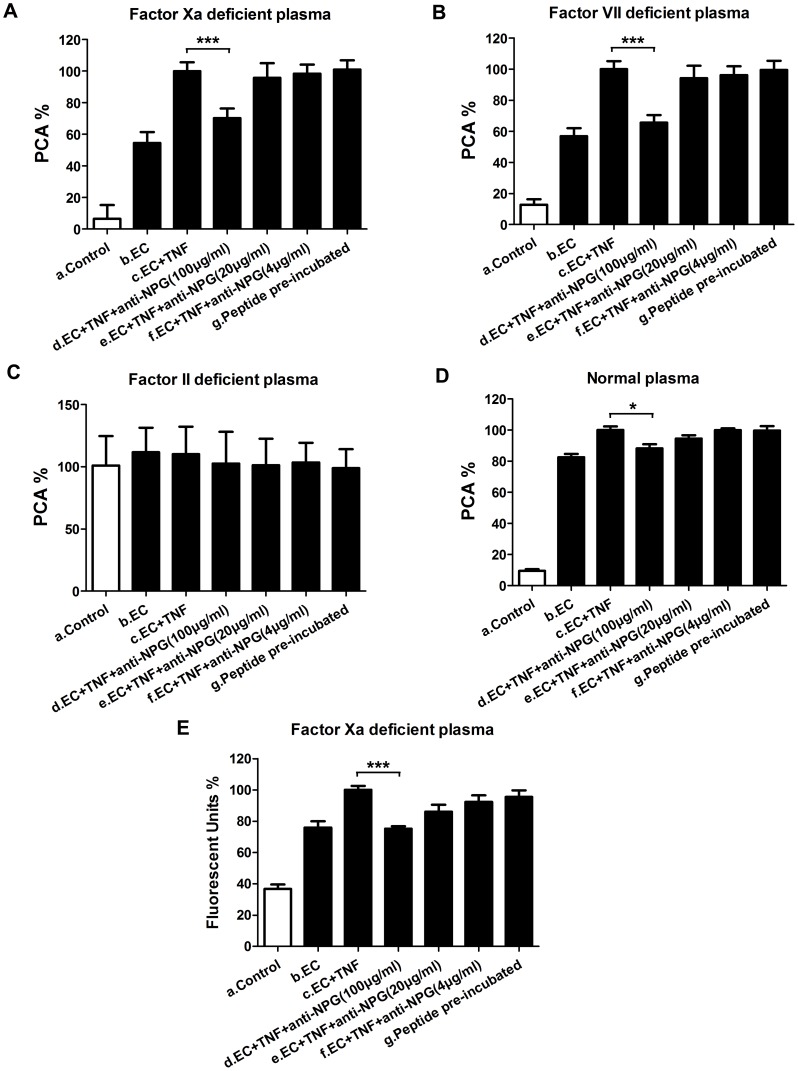
Anti-NPG-12 antibodies exhibited inhibitory effects on prothrombinase activity of hfgl2. (A-D) The fibrin clot generation was evaluated through a one-stage clotting assay in FX, FVII, FII-deficient and normal plasma. The means of PCA units calculated for hfgl2 prothrombinase obtained from the c groups (EC+TNF) were assigned a 100%. The values in the histograms are the means of PCA units obtained from group n/group c ± S.E.M. In figure A and B, group d *vs.* group c, ***P<0.001 (n = 6). In figure D, group d *vs.* group c, *P<0.05 (n = 9). (E) The thrombin generation was evaluated using a Thermo Electron Fluoroskan Ascent fluorometer in FX-deficient plasma. The means of fluorescent units obtained from the c groups (TC+TNF) were assigned a 100%. The values in the histograms are the means of fluorescent units obtained from group n/group c ± S.E.M (n = 3). Group d *vs.* group c, ***P<0.001. EC: HUVECs, TNF: TNF-α, anti-NPG-12: anti-NPG-12 antibodies.

A one-stage clotting assay was used to detect the hfgl2 procoagulant activity (PCA). After marked hfgl2 expression was induced in HUVECs through their exposure to 50 ng/ml TNF-α for 24 h, the PCA% in FX-deficient plasma group increased nearly 50% compared with group b (Fig. 4A). In contrast, the PCA was suppressed by ∼30% when anti-NPG-12 antibodies (100 µg/ml) were added (n = 6, ***P<0.001; Fig. 4A). Similar tendency was observed in FVII-deficient plasma groups (n = 6, ***P<0.001; Fig. 4B) and normal plasma groups (n = 9, *P<0.05; Fig. 4D). No significant difference was observed in FII-deficient plasma groups (Fig. 4C).

A Thermo Electron Fluoroskan Ascent fluorometer was used to detect thrombin generation. The fluorescent units% increased from 38% in the control group to 78% in the HUVEC group compared with TNF-α-pre-stimulated group; the fluorescent units% decreased to 76% when anti-NPG-12 antibodies (100 µg/ml) were added compared with TNF-α-pre-stimulated group (n = 3, ***P<0.001; Fig. 4E).

**Figure 5:**
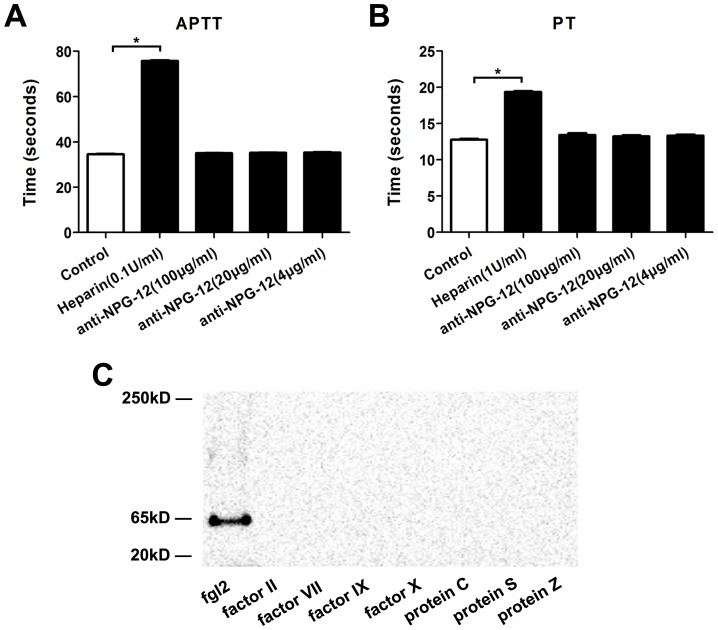
Effect of anti-NPG-12 antibodies on the PT and APTT of human platelet-poor plasma and binding between anti-NPG-12 antibodies and VKD coagulation factors. (A) In APTT test, normal platelet-poor plasma was used as a negative control and platelet-poor plasma with 0.1 U/ml heparin as a positive control. No significant difference was observed among the antibody treated groups *vs* negative control group (n = 3, *P*>0.05). (B) In PT test, normal platelet-poor plasma was used as a negative control and platelet-poor plasma with 1 U/ml heparin as a positive control. No significant difference was observed among the antibody treated groups *vs* negative control group (n = 3, *P*>0.05). (C) In WB test, anti-NPG-12 antibodies combination with hfgl2 expressed by HUVECs at a location of 65 kD was used as a positive control. No detection was observed in the VKD coagulation factor groups. anti-NPG-12: anti-NPG-12 antibodies.

PT and APTT illustrated the clotting time of citrated normal human platelet-poor plasma in the presence or absence of anti-NPG-12 antibodies at different concentrations. The results showed that there were no significant differences among the groups with negative control (n = 3, *P*>0.05; Fig. 5A and Fig. 5B), and heparin was added as a positive control.

VKD coagulation factors contain a γ-carboxyglutamic acid (Gla) domain which is rich in Glu residues [46]. Western Blotting tests were used to detect anti-NPG-12 antibodies' possible cross-reaction with VKD coagulation factors, including factor II, factor VII, factor IX, factor X, protein C, protein S and protein Z [47]. As a positive control, anti-NPG-12 antibodies recognized hfgl2 at a location of 65 kD. In contrast, there was no detection in the VKD coagulation factor groups. The results indicated no detectable cross-reaction between anti-NPG-12 antibodies and VKD coagulation factors.

The experiments described so far provided evidence that induced highly expressed fgl2 took part in nearly 30% of total PCA, and the antibodies' inhibitory effect on hfgl2 prothrombinase activity was dependent on FII and independent on FX or FVII, consistent with previous report [10].

### Influence of anti-NPG-12 antibodies on T lymphocyte proliferation and activation

As anti-NPG-12 antibodies were created against the amino acids 76^th^-87^th^ of fgl2, it would not be expected to bind hfgl2 FRED domain or exhibit inhibition of T cell proliferation or activation. Previous studies have demonstrated that agonist anti-CD3 and anti-CD28 mAbs could bind with T cells, and then provide first signal and second signal to induce T cell proliferation [42], [48]. Thus, to examine influence of anti-NPG-12 antibodies on bulk T cell population, anti-CD3/anti-CD28 mAbs were used to promote T cell proliferation in this study.

**Figure 6:**
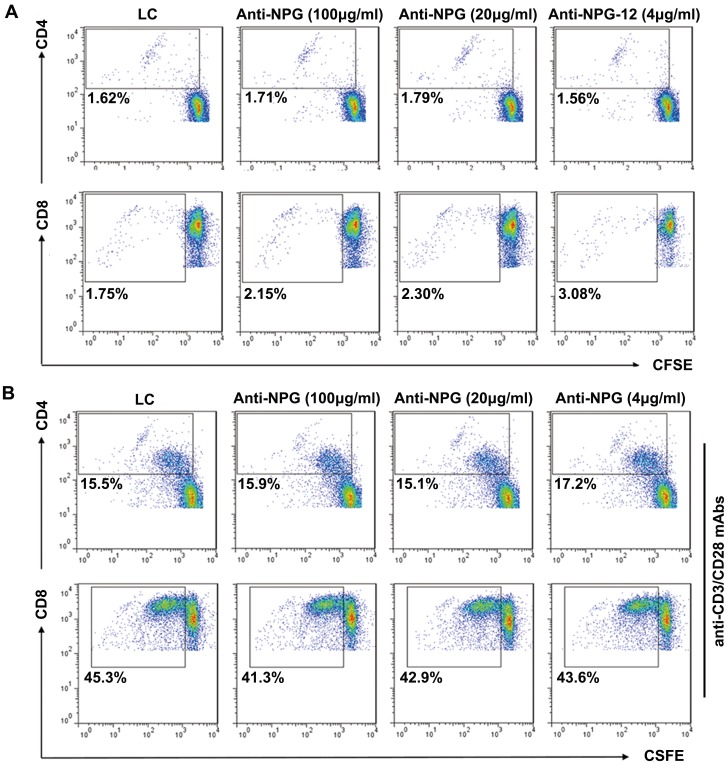
Anti-NPG-12 antibodies exhibited no influence on the proliferation of human peripheral blood T lymphocytes. (A) There was no significant difference in the CD4^+^ and CD8^+^ T lymphocyte proliferation in the absence of anti-CD3/CD28, as determined through FCM analysis (*P*>0.05). (B) There was no significant difference in the CD4^+^ and CD8^+^ T lymphocyte proliferation in the presence of anti-CD3/CD28, as determined through FCM analysis (*P*>0.05).

FCM was used to analyze the proliferation of CFSE-labeled CD4^+^ and CD8^+^ T cells in the presence or absence of anti-NPG-12 antibodies at different concentrations. The results demonstrated that the antibodies could neither promote CD4^+^ and CD8^+^ T cell proliferation (Fig. 6A) nor inhibit their proliferation when the cells were stimulated with anti-CD3/CD28 (2 µg/ml) for 72 h (Fig. 6B).

**Figure 7:**
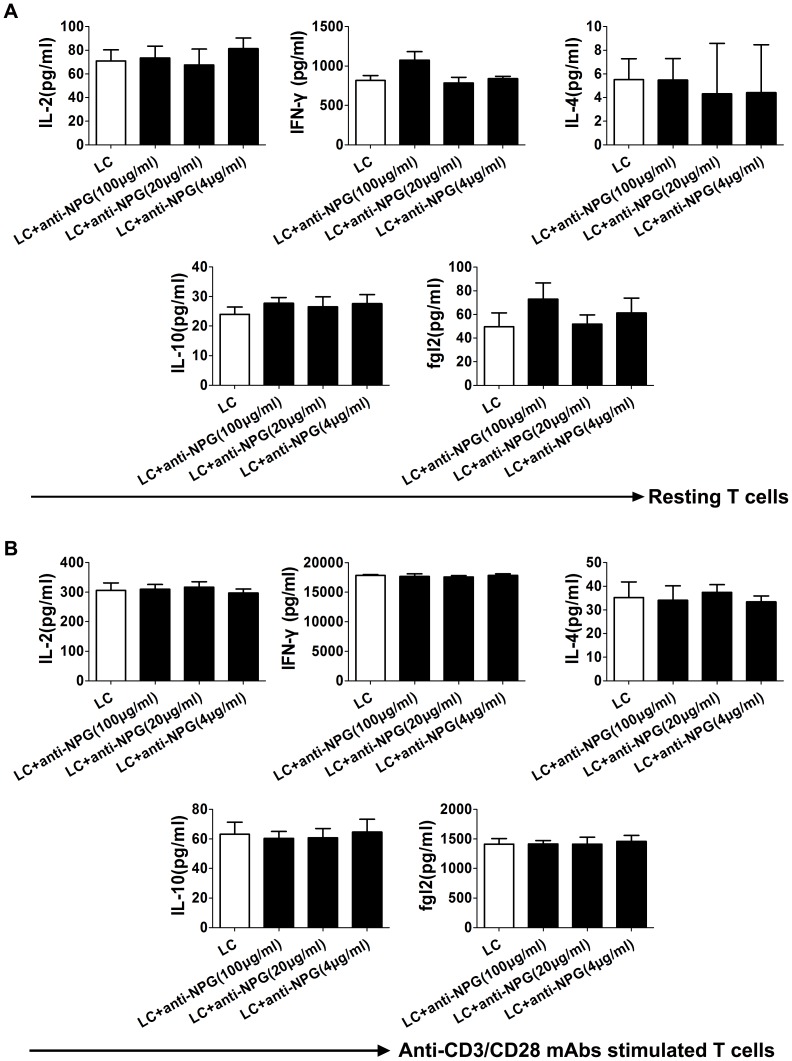
Anti-NPG-12 antibodies exhibited no influence on the polarization of human peripheral blood T lymphocytes. (A) No significant differences were observed in the levels of IL-2, IFN-γ, IL-4, IL-10 and secreted fgl2 in the supernatants from the resting T lymphocytes, as determined by ELISA (*P*>0.05). The values are the means ± S.E.M (n = 3). (B) No significant differences were observed in the levels of IL-2, IFN-γ, IL-4, IL-10 and secreted fgl2 in the supernatants from the proliferated T lymphocytes stimulated by anti-CD3/CD28 mAb, as determined by ELISA (*P*>0.05). The values are the means ± S.E.M (n = 3). LC: lymphocytes, anti-NPG: anti-NPG-12 antibodies.

Double-antibody sandwich ELISA was applied to detect the levels of IL-2, IFN-γ, IL-4, IL-10 and secreted fgl2 in the cultured cell supernatants. No significant differences were observed in the levels of these five cytokines between the groups, indicating that the antibodies, at the tested concentrations, may not alter the differentiation or polarization of CD4^+^ and CD8^+^ T lymphocytes in the presence or absence of anti-CD3/CD28 for 72 h (*P*>0.05; Fig. 7A and 7B).

## Discussion

In this study, we identified a peptide named NPG-12 located at the hfgl2 N-terminal linear coiled-coil domain near Ser91 that contains abundant Glu residues. As a novel and specific hfgl2 inhibitor, anti-NPG-12 antibodies were produced against the antigenic determinant NPG-12 and were able to inhibit hfgl2 PCA and hfgl2-induced thrombin generation without influencing the PT and APTT, or exerting detectable influence on CD4^+^ and CD8^+^ T lymphocyte activation.

Although hfgl2 was shown to be a serine protease by DFP inhibition, classical serine peptidase reaction site as Ser195-His57-Asp102 catalytic triad was not found in its sequence [10], [49]. Instead, four potential serine peptidase clan E catalyzing sites (SXXK) were found at positions 45, 91, 142, and 423 (Fig. 2). On the other hand, γ-Carboxyglutamic acids (Gla) formed by Glu γ-carboxylation can give rise to an affinity for Ca^2+^ which is a prerequisite step in the coagulation pathway [10], [13], [50], [51]. Calcium-rich Gla was proposed to be responsible for adherence to the phospholipid backbone and Factor Va [10], [51], [52]. Thus, we hypothesize that the prothrombinase activity of hfgl2 serine protease may be dependent on the SXXK clan SE catalyzing site and a Glu-rich domain. There are thirty-two Glu residues exist in the hfgl2 sequence; particularly, 12 of which scatter between positions 26 and 87 and nine between positions 124 and 170. Levy et al. revealed that a mutation at Ser135 of mfgl2 has no impact on the prothrombinase activity of the protein, whereas Ser89 of mfgl2 plays a crucial role in its prothrombinase activity [10]. These findings led us to look closely at the polypeptide sequences mapped to the 76^th^-87^th^, 49^th^-56^th^, and 26^th^-44^th^ residues near hfgl2 Ser91, which are with acceptable hydrophilic and antigenicity determined by HomoloGene and DNAstar (Fig. 2) [2], [10], [20], [23]. Moreover, because all these peptides are located in the extracellular domains and molecular surface of membrane-bound hfgl2, theoretically it will be easier for antibodies to bind them through direct interaction.

Ser89 is a critical site for the prothrombinase activity of mfgl2 and rat fgl2 [10], [13], [50]. In a previous study, our group demonstrated that rat fgl2 is highly expressed in association with microthrombosis in rat type 2 diabetes and cardiac ischemia/reperfusion models [7]–[9]. We identified two rat fgl2 peptide sequences (Pep1 and Pep2) rich in Glu residues that exhibit acceptable hydrophilic and antigenicity. Pep2 (74^th^ to 85^th^) is proximal to Ser89, whereas Pep1 (41^st^–52^nd^) is distal from Ser89. Both antibodies against Pep1 and Pep2 exhibited specificity and anti-coagulant activity. Nevertheless, the anti-Pep2 antibodies possessed a stronger affinity to rat fgl2 and more intensive inhibition on rat fgl2 prothrombinase activity, as evidenced by cultured rat microvascular endothelial cells in vitro or rat cardiac ischemia/reperfusion model in vivo (unpublished data). These findings suggest that the special distance between target peptide and the Ser89 site may have a negative correlation to inhibitory effect of antibodies on the fgl2 prothrombinase activity. Therefore, NPG-12, which is near Ser91on hfgl2 and similar to rat fgl2 Pep2, seems to be an excellent target.

To examine the effectiveness of the anti-NPG-12 antibodies, we induced a high expression of hfgl2 in HUVECs by treating the cells with TNF-α, as previously described in cultured PAECs [2], [7], [29], [53].We confirmed the specificity of the anti-NPG-12 antibodies by ELISA, Western blot and immunostaining (Fig. 3). In further experiments, the anti-NPG-12 antibodies exhibited evident anti-coagulation effects through the suppression of the procoagulant activity and thrombin generation of hfgl2 in Factor Xa deficient plasma while exerting undetectable influence on the classic intrinsic and extrinsic coagulation pathways, as indicated by the PT and APTT (Fig. 4–5). These results demonstrate a direct targeting of the hfgl2 prothrombinase activity without cross-reaction to VKD coagulation factors (Fig. 4–5). As shown in Fig. 4D, the PCA was suppressed by ∼10% when anti-NPG-12 antibodies (100 µg/ml) were added (n = 9, *P<0.05). However, fibrin deposit could be suppressed by ∼50% in a rat model interfered by anti-rat-NPG-12 antibody (data not shown). We speculated that it was the result from differences between *in vivo* and *in vitro* models and the results indicated that in HUVECs tissue factor-Factor VII classic coagulation pathway may play a more important role than fgl2 coagulation pathway in normal plasma. To rule out as much cross-reaction to other Ser-containing prothrombinases as possible, we chose to target the Glu residues for Ca^2+^ binding near Ser91XXK, which may be essential for the acceleration of the coagulation [10], [13], [54]. Thus, it is worth noting that the spatial structure of the anti-NPG-12 antibodies may not affect other serine proteases because hfgl2 NPG-12 does not share any antigenic determinants with other prothrombinases. We also put emphasis on domain selectivity when designing NPG-12, as it was chosen from outside the FRED fragment in order to minimize both cross-reaction with fibrinogen and influence on CD4^+^ and CD8^+^ T lymphocyte proliferation and polarization (Fig. 1, Fig. 6 and Fig. 7). This should be taken into consideration because the FRED fragment shows high homology with the β- and γ- chains of fibrinogen and immunomodulatory activity in regulating the proliferation and polarization of T lymphocytes [16], [42].

**Figure 8:**
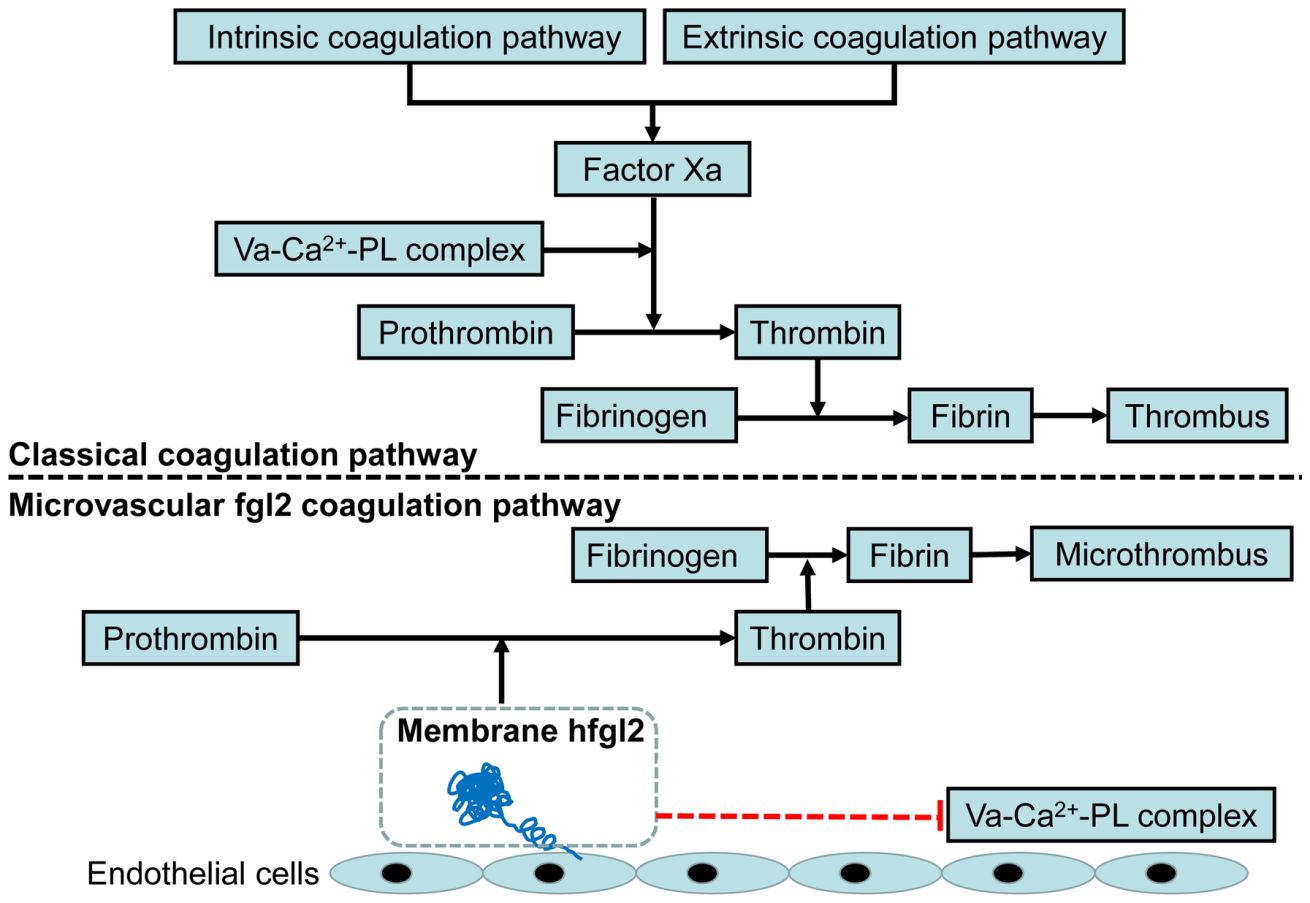
A possible mechanism underlying the fgl2 coagulation pathway and the inhibition of fgl2 prothrombinase activity. Based on our observations and the results from other groups, the possible mechanism underlying the fgl2 coagulation pathway and inhibition of NPG-12 are summarized. Va: factor Va, PL: phospholipids, ECs: endothelial cells.

Several lines of *in vivo* evidences indicate that fgl2 serves as a multifunctional protein [11], [55]. In addition, it has been reported that fgl2 may be a more important procoagulant than tissue factor in xenograft acute vascular rejection characterized by fibrin deposition *in situ* or microvascular thrombosis [11], [56]. These findings may explain why anticoagulants against the classic coagulation pathway can effectively ameliorate macrovascular thrombus but are not as efficient to prevent the formation of microvascular thrombus associated with microcirculatory disturbances (Fig. 8) [56], [57].

In conclusion, we have successfully produced novel polyclonal antibodies against NPG-12 that can inhibit hfgl2 prothrombinase activity. Our findings indicate that NPG-12 may serve as a critical domain for hfgl2 prothrombinase activity. Monoclonal antibody against NPG-12, human fgl2 transgenic mice or fgl2 knockout mice could be illuminating for the research or management of membrane-bound hfgl2-associated microcirculatory disturbances. Moreover, genetic diversity of the fgl2 region of interest, and next generation sequencing and development of inhibitors against this domain could be undertaken in future.
